# Covid-19: Early Cases and Disease Spread

**DOI:** 10.5334/aogh.3776

**Published:** 2022-09-29

**Authors:** Jacques Reis, Alain Le Faou, Alain Buguet, Guy Sandner, Peter Spencer

**Affiliations:** 1 UDS University of Strasbourg, 67000 Strasbourg, France; 2 Association RISE, 67205 Oberhausbergen, France; 3 EA 3452 CITHEFOR and Faculté de Médecine Maïeutique et Métiers de la Santé. Université de Lorraine 54500 Vandoeuvre-lès-Nancy, France; 4 Malaria Research Unit, UMR 5246 CNRS, Claude-Bernard Lyon-1 University, 69622 Villeurbanne, France; 5 Faculty of medicine, University of Strasbourg. 6 rue de Neufeld, 67100 Strasbourg, France; 6 Department of Neurology, School of Medicine, and Oregon Institute of Occupational Health Sciences, Oregon Health & Science University, Portland, OR 97239, USA

**Keywords:** Primary case, index case, Wuhan, Military World Games, retrospective assessment methods, early SARS-CoV-2 circulation

## Abstract

The emergence and global spread of the Severe Acute Respiratory Syndrome Coronavirus-2 (SARS-CoV-2) is critical to understanding how to prevent or control a future viral pandemic. We review the tools used for this retrospective search, their limits, and results obtained from China, France, Italy and the USA. We examine possible scenarios for the emergence of SARS-CoV-2 in the human population. We consider the Chinese city of Wuhan where the first cases of atypical pneumonia were attributed to SARS-CoV-2 and from where the disease spread worldwide. Possible superspreading events include the Wuhan-based 7^th^ Military World Games on October 18–27, 2019 and the Chinese New Year holidays from January 25 to February 2, 2020. Several clues point to an early regional circulation of SARS-CoV-2 in northern Italy (Lombardi) as soon as September/October 2019 and in France in November/December 2019, if not before. With the goal of preventing future pandemics, we call for additional retrospective studies designed to trace the origin of SARS-CoV-2.

## Introduction

In February 2020, The World Health Organization (WHO) officially declared the first confirmed Covid-19 case (index case) in China as having occurred on December 8, 2019, following an official statement from the Chinese Government [1]. Investigation on how and when the Covid-19 pandemic began was recommended by the WHO in March 2020 [2]; this was followed by the WHO-China Working Group [3] and an international scientific committee [4]. In response to a May 2020 World Health Assembly, the WHO Director-General announced the establishment of a Scientific Advisory Group for Origins (SAGO) of Novel Pathogens on July 14 [5,6]. A panel of 26 scientists was selected (www.who.int/director-general/speeches/detail/who-director-general-s-opening-remarks-at-the-media-briefing-on-covid-19-13-october-2021) for this purpose.

Debate on the ‘origin’ of the Severe Acute Respiratory Syndrome Coronavirus-2 (SARS-CoV-2) is blurred by ideology and geopolitical issues [7]. We use the word ‘origin’ in reference to Wolfe and colleagues’ question ‘Where did they come from?’ in their review on the origin of major infectious diseases [8]. The ‘origin’ of some major ‘historical’ human infectious diseases (e.g., tuberculosis, cholera) remains speculative [8], and the real source of some well-known recent viral epidemics is often only suspected (e.g., human Coronaviruses) [9]. Furthermore, the respective role of ecosystem disruption in optimizing conditions for transfer of viruses to humans, and the existence of reservoirs and intermediate hosts is a complex issue that is not always resolved. The ‘One Health’ approach would be helpful for solving this problem [10].

The objective of this review is to describe what is known about events relating to the emergence of Covid-19: the first early cases, and how and when they were diagnosed. The hypotheses for the emergence of the SARS-CoV-2 pathogen are evoked as an incentive to go further in these investigations. Understanding how and why SARS-CoV-2 emerged in the human population and was able to spread globally will guide efforts and identify tools to control or prevent a future viral pandemic [2,3,4]. In the case of human-to-human transmission of an infective agent, the first person identified with the pathogen (primary case) who transmits the infection to others in a community [11] is seldom identified and not necessarily ill. The spread on an infective agent refers to various concerns, such as its characteristics (contagiousness), its transmission routes classified in interhuman (direct) or indirect (e.g., via fomites), the role of physical and chemical environmental conditions for its germ survival, and the human-to-human transmission that can be mathematically modelled (stochastic transmission in the case of SARS-CoV-2). Although having reviewed these concerns for SARS-CoV-2 recently [12], new data and analyzes are considered.

## Why is it difficult to obtain this information?

The epidemy of respiratory disease in Wuhan, Hubei province, China, triggered the rapid identification and characterization of the causal coronavirus (SARS-CoV-2) by sequencing its genomic RNA and devising diagnostic molecular techniques. The index case of Covid-19 [11] was officially designated as a patient admitted to hospital in Wuhan City on December 8, 2019 [1]. Whether sick patients with the same clinical and diagnostic manifestations of Covid-19 were present in the region before this date remains unknown. Since the actual origin of the virus is not formally established, the only way to know if the disease had spread prior to December 2019 is to survey data not only from Wuhan but also from elsewhere worldwide. Such retrospective studies are difficult and cumbersome since they depend on the availability of medical files and biological samples (e.g., blood, nasopharyngeal and oropharyngeal swabs specimens, bronchoalveolar lavages) in diverse hospitals and institutions.

## Which tools can be used and what are their limits?

- RT-PCR detection: This sensitive and accurate method of analysis requires samples preserved in good condition (i.e., storage at –80∞C), which is seldom the case. Frozen samples are not suitable for cell culture (on Vero cells), and this method is less sensitive than RT-PCR even for fresh samples [13]
- Serologic studies: Diagnostic serological tests can be used to screen patient sera obtained from blood banks. This is a useful approach, provided there is no cross-reactivity with human endemic coronaviruses (HcoV-229E, HcoV-NL63, HcoV-HKU1, HcoV-OC43) responsible for common colds and seldom pneumonia [14]. This may explain reports of positive serology results well before pre-pandemic periods [15].
- Phylogenetic studies: These sequence and compare the genomes of available strains and use the data to try to identify the first putative virus isolate as well as the date of its emergence [16]. Unfortunately, this approach is of limited value for coronaviruses because of -post-transciptional genome editing (transitions C => U) that adds to an already high rate of mutations [17]. However, despite these uncertainties, such studies are in favor of an earlier beginning of the pandemic, between July and Fall 2019 [18,19,20,21,22,23,24]. These findings must be interpreted with caution, especially given that the first available complete genome sequences were obtained in January 2020.
- Detection of viral RNA in sewage: SARS-CoV-2 infects intestinal epithelium and is thus eliminated in feces either in free form or inside detached enterocytes, which ensures that the viral genome is present in waste waters and may be detected in sewage. This provides an indirect method of virus surveillance to track community infection in space and time. Several teams have used this ‘wastewater-based epidemiology’, notably in Italy [25], Spain [26], and Brazil [27]. However, it should be noted that SARS-CoV-2 RNA is a fragile molecule readily degraded by ubiquitous ribonucleases, which renders the results of retrospective studies on preserved samples uncertain. Additionally, given the sample dilution during wastewater collection and treatment, this research technique may have limited value in detecting early cases given that the community caseload would be small [28].
- Computed tomography (CT): CT images of the lungs of patients with Covid-19 [29] have high sensitivity (90%) but moderate specificity (61%) [30]. Chest CT was the first diagnostic tool used for Covid-19 patients and preceded the availability of specific laboratory tests [31,32,33]. While chest CT provided a reasonably good diagnostic tool, in an epidemic situation it is barely usable for the retrospective identification of infected patients prior to the index case because there was presumably a low prevalence of SARS-CoV-2 infection; thus, the imaging technique has in this case a poor positive predictive value.
- Retrospective clinical approach: This has many pitfalls because the initial spread of SARS-CoV-2 is probably attributable in large part to persons who were asymptomatic, had mild symptoms, or ones that mimicked common respiratory infections. Another caveat is related to overtly symptomatic patients who were misdiagnosed because of an atypical clinical presentation given that Covid-19 is a multisystemic disease [34,35,36,37,38]. For example, clinicians at the beginning of the pandemic would have assumed that Covid-19 symptoms mostly matched those of the common cold or influenza, whether an atypical pneumonia was absent or present. From a public health perspective, such diagnostic failures compromised the accurate estimation of Covid-19 parameters (notably incidence).
- Epidemiological surveys: These rely on a range of techniques, such as retrospective patient/community questionnaires, review of clinical data, and hospital laboratory results confirming pneumonia of unknown origin. At best, the results of such studies lack specificity and render interpretation difficult, although they may provide suspicion of Covid-19 cases.

Altogether, even if clinical and epidemiological data can give a hint of SARS-Cov-2 infections, positive diagnostic certainty can only be obtained using molecular diagnostic techniques.

## What are the certainties and hints of cases prior to the first official Covid-19 case?

### From public media

Although Covid-like symptoms were reported in several journals or as oral communications, none has given rise to a published medical or scientific report, which is regrettable. Since none of the proposed cases had laboratory confirmation, their value must be questioned (Insert 1 and 2). However, we consider that such news items cannot be simply dismissed.

### From surveys or retrospective investigations: RT-PCR and Serology

Valuable studies have been conducted in several countries that confirm the presence of the virus at the time of the beginning of the epidemic in Wuhan and even before.

### France

Officially, the first Covid-19 case in Europe was diagnosed in France on January 24, 2020. This patient and the following four cases had visited Hubei province or arrived directly from Wuhan [39,40,41]. The first biologically confirmed French case of Covid-19 dates from 27 December 2019, a patient with an influenza-like illness. The diagnosis was obtained retrospectively by RT-PCR performed on stored respiratory samples, while the ones collected from 58 patients hospitalized in an ICU for pneumonia from December 2, 2019 to January 16, 2020 gave negative results [42]. An epidemiological study was performed in Alsace before February 26, 2020, the date on which the first official case of Covid-19 was recognized in the region. This study relied on three independent and retrospective sources: a population-based survey, an analysis of medical records from hospital emergency-care services, and data from the medical biology laboratories of a private hospital. All these sources point to a beginning of community SARS-CoV-2 infection at the end of January 2020, one month before the first diagnosed case of Covid-19 [43]. Testing of serum samples routinely collected from November 4, 2019 to March 16, 2020 from 9,144 adults included in CONSTANCES, a randomly selected general-purpose cohort of French adults aged 18–69 years at recruitment, yielded 13 positive antibody test results (anti-SARS-CoV-2 IgG) in blood samples taken from November 5, 2019 to January 30, 2020. Of these, 11 participants were interviewed; six did not report symptoms during the week preceding the sampling. This study is in favor of SARS-CoV-2 infections in France as early as November 2019 [44].

### Italy

In Italy, the first notified cases corresponded to two Chinese tourists from Wuhan who were hospitalized in Rome’s Spallanzani Hospital on January 31, 2020 and who were likely infected before their arrival [45]. Nonetheless, the first autochthonous (index case) was notified on February 21, 2020 in Codogno, province of Lodi, Lombardy [46]. However, the first confirmed clinical case was sampled on November 21, 2019; he was a four year-old Milanese boy who was hospitalized with a morbilliform rash and thus a suspicion of measles. SARS-CoV-2 infection was diagnosed retrospectively by RT-PCR analysis of stored oropharyngeal samples; this was the only positive result obtained among 39 other specimens collected from September 2019 to February 2020 from consenting patients who had presented with a similar non-measles-linked rash [46]. Medical records of the emergency department of the Fondazione Ca’ Granda Policlinico, a downtown Milan hospital, showed an increase in the number of patients complaining of fever, cough and dyspnea between January 11 and February 15, 2020, as compared to the three previous years [47]. Serological testing of samples of donated blood that were collected between January 27 and February 20, 2020 in the Lodi province [48] and from February 24 to April 8, 2020 in Milan [49], found positive antibodies against SARS-CoV-2 in five sera for the former and 36 for the latter. By contrast, in Rome, there was no evidence of SARS-CoV-2 circulation before the date of the official beginning of the outbreak of Covid-19 in Italy [50].

The testing of a sample from a cohort of 959 asymptomatic individuals enrolled in a prospective lung-screening trial across Italy between September 2019 and March 2020 revealed 111 positive cases (11.6%) for SARS-CoV-2 receptor binding domain-specific antibodies. Fifty-nine subjects were residents of Lombardy. The first positive results dated back to September 2019 [51]. Positive RT-PCRs were found among samples of water-treatment plants; these were collected between October 9, 2019 and February 28, 2020, in Milan and Turin on December 18, 2019 and in Bologna on January 29, 2020 [52]. A phylogenetic study of 346 SARS-CoV-2 complete viral genomes obtained from nasopharyngeal swabs of hospitalized patients revealed two lineages (clades B and A.2) and concluded that a sustained community transmission was underway before the first Covid-19 case was detected in Lombardy [53].

Taken together, these findings suggest SARS-CoV-2 was circulating regionally in northern Italy (Lombardi) as early as September/October 2019, at least one month before the virus was detected in France.

### USA Mainland

In the USA, 12 cases of SARS-CoV-2 infection were registered in January 2020, of which 10 came from China and two had had contact with them [54]. The first American patient returning from Wuhan was hospitalized on January 20, 2020 [55]. The first autochthonous case, not directly related to the former patients, was diagnosed on February 26, 2020 in California [56].

An earlier presence of SARS-CoV-2 in the United States was suspected based on the results of testing of sera obtained from routine blood donations to the American Red Cross. Of 7,389 samples analyzed, some of which had been collected between December 13 and 16, 2019, 106 (1.44%) were positive [57]. On the East coast, the first Covid-19 case was diagnosed in New York City on February 29, 2020 [58]. Based on phylogenetic reconstructions, it has been estimated that independent SARS-CoV-2 introductions had occurred no later than early February and potentially as soon as January 8, 2020 [59].

### China

After an initial hesitation, the scientific and medical communities in China reacted very rapidly at the beginning of the epidemic in Wuhan. Chinese scientists grew SARS-CoV-2 in cell culture, sequenced its genome and developed a molecular diagnostic test. Nevertheless, Li and colleagues [60] were aware of the limitations of their study of 425 cases, as molecular testing for SARS-CoV-2 infection only became available in Wuhan on January 11, 2020. No information has been publicly available on any community/patient testing prior to the first official case (December 8, 2019), although the March 13, 2020 edition of the Hong Kong-based *South China Morning Post* reported a previous case that has not been confirmed by the authorities (see box).

A serological study of SARS-CoV-2 infections was conducted in Wuhan pet and stray cats, animals easily contaminated by the virus. Animal blood samples collected in March-May 2019 were negative while those sampled in the first quarter of 2020 were positive [61].

A Harvard university study [62] claimed there was an increase of traffic in Wuhan hospitals and of patients with diarrhea in late Summer and Fall of 2019. This study has major limitations and uncertainties. Diarrhea, for which a compilation of queries via the Baidu search engine was conducted, is not a common symptom of Covid-19 [63,64]. Moreover, this study did not consider the possibility that diarrhea cases and increased hospital traffic were associated with the region’s catastrophic floods of July 2019 (www.voanews.com/a/east-asia-pacific_heavy-rain-flooding-china-force-evacuation-nearly-8/6171630.html).

In summary, the different results obtained from the three occidental countries conservatively indicate that cases of Covid-19 were already present by the end of December 2019 and possibly somewhat earlier, a conclusion with which Chinese researchers agree [65]. This favors the proposal that the Covid-19 epidemic began at least as early as the last quarter of 2019.

## What are the possible scenarios for the emergence of SARS-CoV-2 in the human population?

- Viral transmission from an animal directly or indirectly is favored by epidemiologists given that such events have occurred several times in the recent past [66]. Bats, which harbor numerous coronavirus strains, are designated as the most likely culprit [67]. Direct infection, or infection via an intermediate animal host infection, are both possible [68].
- The occurrence of a human coronavirus that has evolved in humans and has acquired pathologic potential is rather unlikely since examples are lacking even though some ‘commensal’ viruses have been implicated in diseases; this is always an uncommon event, sometimes related to immunodepression (e.g., JC virus and progressive multifocal leucoencephalitis).
- A mixed infection of a (human) non-pathogenic coronavirus and an outlier would give birth to a recombinant virus (a common phenomenon among the coronaviruses) having acquired a pathogenic ability from the second strain (e.g., the gene coding a spike gene that recognizes the ACE-2 receptor) with adaptability to humans provided by the first strain.
- Escape of a strain from a laboratory might occur. Handling the strain might accidentally contaminate a staff member, who then infects his surroundings. Less likely would be a faulty handling of contaminated, non-sterilized material that was disposed of in garbage or effluents (air, water). Intentional manipulation of a strain to make it highly infectious and/or pathogenic for humans has been suggested, but there is no evidence to support such an hypothesis [69].

Whatever the route of human contamination, the acquisition of a ‘new’ virus may lead to different events:

- If the incoming virus is not able to pass to another human, the infected person will develop symptoms but no related case will usually be observed [70]. If the virus is transmitted among humans, this may trigger an epidemic, even a pandemic. This would be limited in the absence of efficacious inter-human transmission, such that viral spread would disappear if appropriate prophylactic measures are taken, as exemplified by the SARS-CoV-1 pandemic from November 2002 to July 2003 [71].
- In the case of SARS-CoV-2, this coronavirus should have acquired an ability to multiply in humans, to generate symptoms and, contemporaneously, to pass readily from one individual to others. With time, and after several passages, the virus would have gained in transmissibility and pathogenic potential, whereupon the conditions for a severe pandemic were fulfilled.
- A succession of infections by different (closely related) viruses may have occurred, but only one was selected to become a human strain, with its airborne transmission facilitating its expansion.

Factors that may have favored the pathogenic evolution of SARS-CoV-2 in vivo and the initiation of an epidemic include:

- Overcrowding, and the rapid passage from one individual to another;
- High viral load in bodily secretions, which facilitate pathogen transmission;
- Travel by infected individuals (asymptomatic at this time) from a contaminated to a ‘naive’ region;
- Immunocompromised individuals in whom a virus can continuously replicate, acquire mutations, and gain resistance to immune defenses and antiviral treatment [72], and eventually develop a pathogenic potential that allows it to be transmitted to the general population.

While the airborne transmission of SARS-CoV-2 is certain [73,74,75], and well defined, (www.who.int/news-room/questions-and-answers/item/coronavirus-disease-covid-19-how-is-it-transmitted), Chinese scientists have also proposed that the coronavirus may be transmitted from food or other packages transported frozen from origin to destination via a cold chain [65,76]. Even unfrozen seafood caught off Xiamen were required to undergo SARS-CoV-2 screening in August 2022 (https://www.bbc.com/news/world-asia-china-62593217). After having reviewed the persistence of SARS-CoV-2 on surfaces and various clues about its survival, Baker and Gibson have definitively refuted the foodborne transmission, underlining the probable oral transmission (aerosol and droplet) between food-related workers [77]. The risk of transmission by aerosols or surface water contamination from poorly treated wastewater (in which SARS-CoV-2 RNA has been detected) has been addressed, notably in rural areas and low- and middle -income countries. This concern is still scrutinized [78].

## Was Wuhan the epicenter of the pandemic?

Wuhan became known worldwide after the discovery of the first atypical pneumonia cases attributed to SARS-CoV-2 in 2019, later named Covid-19. Known as the “Gateway to Nine Provinces” because the city serves as a major commuting center in China, Wuhan is included in the ‘sponge cities’ due to its orographic situation and its climatic vulnerability to floods [79]. Thus, Wuhan was hit in the Summer 2019 by severe floods (https://www.sixthtone.com/news/1004168/after-heavy-rains%2C-flood-control-fears-float-to-the-surface). Their impact on ecosystems as well as their health consequences, water-borne contamination, increase of transmissible diseases, gathering of people in common public facilities favoring virus transmission, should be considered. That Wuhan experienced conditions optimal for the disease to emerge and transmit among humans is a reasonable but hypothetical conjecture [80]. Until now, the question as to whether the location in which the virus was first reported (Wuhan) was necessarily the site of its origin remains unsolved [6,65]. Animal-to-human contact in the Huanan Wholesale Seafood Market of Wuhan has been held responsible for the official index case of Covid-19, but whether this was the place of origin of the pandemic was rapidly questioned [81].

## Which facts may explain the rapid spread worldwide?

The preceding observations show that SARS-CoV-2 was circulating in some Western countries even before the first official case was declared in Wuhan. Thus, if one admits circulation of the virus before this date, its spread to countries beyond China (assuming the virus really originated in this country) was almost concomitant. An event that may have contributed to the dissemination of SARS-CoV-2 is the 7^th^ World Military Games, which was held in Wuhan between October 18 and 28, 2019. More than 10,000 athletes, plus staff from 110 countries, gathered in designated areas (www.xinhuanet.com/english/2019-10/26/c_138505690.htm). No official information has been made available despite reports that some foreign participants experienced Covid-19-compatible symptoms that were attributed to influenza or gastroenteritis. Although three articles evoked this hypothesis [82,83,84], one can only rely on American and European media reports as addressed in supplementary information Insert 3.

Travelers from and to China may have introduced the virus to several countries, notably France, Italy and the USA, either as tourists or for business. For example, the tight economic links between Lombardy and China might have contributed to the early Covid-19 epidemic in this region before the virus and associated disease spread to other parts of Italy [85,86]. The relationship between commercial trade and the SARS-CoV-2 spread was examined in Italy [87], European countries [88] and at a planetary level [89], showing that international trade should be considered as ‘*one of the main indicators of the COVID-19 spread*’ [89].

The Chinese New Year national holiday between January 25 to February 2, 2020, when people travelled to visit family at home and abroad, is considered to be a SARS-CoV-2 superspreading event. Thus, as early as January 2020, members of the International Society for Travel Medicine warned of the potential threat posed by infected people on board international flights from China, and in particular from Wuhan, pointing to airport hubs they considered to be at high-risk for pathogen transmission [90,91].

Social gathering also favors cross-infection and allows a pathogen to travel. This happened in Mulhouse, in the Alsace region of France, where a religious meeting took place on February 17–21, 2020. The meeting was attended by about 2,500 participants. This apparent ‘superspreading event’ has been held responsible for a subsequent dissemination of SARS-CoV-2 across continental France, Corsica and French Guyana [43,44], a remarkable dissemination that, unfortunately, did not give birth to any epidemiological or biological study.

## Conclusions

This review highlights the importance of continued research efforts to track the origin, emergence, regional and global spread of SARS-CoV-2 that resulted in the Covid-19 pandemic. A comparison with what occurred with the most closely related virus, SARS-CoV-1, which arose in Guangdong, China, and spread to 32 countries or regions, might help to understand why only SARS-CoV-2 has become so well adapted to a human host. Such investigations are needed to design strategies for the prevention, rapid detection, early response and management of new infective strains of SARS-CoV-2 and other viruses that can threaten human health on such a large scale.

For the moment, according to several reports, it is known that SARS-CoV-2 was present in countries outside China in the last quarter of 2019 but crucially important information for this period from mainland China is lacking. What happened in China is still unsolved and controversial. Recent studies (preprints), again implicate the Huanan market as the place where the pandemic started and offer the hypothesis of at least two independent introductions of SARS-CoV-2 into the Wuhan population [92,93]. These preprints were published in *Science* in Summer 2022 [94,95]. One stated, even: ‘*These findings indicate that it is unlikely that SARS-CoV-2 circulated widely in humans before November 2019 and define the narrow window between when SARS-CoV-2 first jumped into humans and when the first cases of COVID-19 were reported*’ [95]. It is noticeable that Chinese surveillance of SARS-CoV-2, conducted after the closure of the Huanan market (early 2020), showed the absence of any virus detection (RT-qPCR and culture) in a broad diversity of animals tested while it was present in the environment (stalls in the Western zone) [96]. The role of the market as the origin of the spreading of the virus, although it may have been imported there from elsewhere by an employee or a visitor, leaves unresolved the actual source of this pathogen. This justifies the position of the WHO Advisory Group for the Origins of Novel Pathogens which stated in June 2022: ‘*the source of SARS-CoV-2 and its introduction into the market is unclear and it is yet to be determined where the initial spillover event(s) occurred*’ [6]. Its role as a virus amplification location due to the high number of visitors is also hypothesized [96]. By extension, it would seem reasonable to consider that SARS-CoV-2 made multiple early escapes from China, including to Europe and the USA.

An important initiative would be to seek evidence of the presence of the culpable virus from blood collected at the Wuhan Blood Center during the second half of 2019 (www.businessinsider.com/china-testing-thousands-wuhan-blood-samples-covid-19-origins-2021-10?r=US&IR=T). Unfortunately, as of August 2022, no data have been published. Given the likelihood that drug screening was performed on Chinese and foreign athletes participating in the Wuhan 7th Military World Games, this provides a second opportunity to conduct broad viral screening, provided the blood samples were retained and preserved under suitable conditions. Such studies will need to overcome the geopolitical issues that have mudded scientific research into the origin of SARS-CoV-2, a challenge which if accepted promises to benefit the whole of Humanity [7]. Besides the Chinese conundrum, the emergence of COVID-19 also questions some enigmas in Western reports. As an example, the French ‘Ordre des médecins’, a professional, administrative and jurisdictional organization for the defense and regulation of the medical profession, in close relations with the French administration, was alerted in November 2019 about a China infection outbreak that was later named SARS-CoV-2 Covid-19. An article published in April 2022 in its official bulletin reported this important information [97].

## Additional File

The additional file for this article can be found as follows:

10.5334/aogh.3776.s1 Inserts. Insert N1 to N3 Covid and Web.

## Author Note

Published September 14, 2022, The Lancet Commission on lessons for the future from the Covid-19 pandemic https://www.thelancet.com/journals/lancet/article/PIIS0140-6736(22)01585-9/fulltext describes the multiple failures of international cooperation that promoted the global spread of SARS-CoV-2 and makes recommendations designed to strengthen global public health to prevent and respond to newly emerging diseases. Commissioners held diverse views about the relative probabilities of the proximal origin of SARS-CoV-2 — from zoonotic spillover or from human field or laboratory activity — stating that both possibilities require further scientific investigation.
