# Supplementary material for: Covid-19: Early Cases and Disease Spread
Source: Ann Glob Health. 2022 Sep 29;88(1):83. doi: 10.5334/aogh.3776 (PMC9524236; doi:10.5334/aogh.3776)
Supplement: Inserts. — Insert N1 to N3 Covid and Web. [file agh-88-1-3776-s1.zip › s1-agh-3776_reis/Insert_1.docx]

**Insert N1 Covid and Web. The early occurrence of cases in France and a later superspreading event reported by general media**

Several pieces of information issued from French general media in May 2020 without any confirmation, e.g., publications in scientific journals pointing to an early occurrence of COVID-19 cases in fall 2019 (1,2) and the Alsatian superspreading event in March 2020 (3).

1.[www.fondation-diaconat.fr/images/Presse/2020/CP-HAS-Imagerie-mdicale-7-mai-2020.pdf](https://www.fondation-diaconat.fr/images/Presse/2020/CP-HAS-Imagerie-mdicale-7-mai-2020.pdf). . Access online March 11, 2022

2.www.letelegramme.fr/local/cotes-d-armor/covid-19-une-costarmoricaine-aurait-ete-contaminee-fin-novembre-en-france-15-05-2020-12552890.php. Access online March 11, 2022

3 www.leparisien.fr/societe/au-coeur-des-clusters-a-mulhouse-le-rassemblement-evangelique-qui-a-fait-basculer-la-france-09-05-2020-8313693.php. Access online March 11, 2022
