# Supplementary material for: Covid-19: Early Cases and Disease Spread
Source: Ann Glob Health. 2022 Sep 29;88(1):83. doi: 10.5334/aogh.3776 (PMC9524236; doi:10.5334/aogh.3776)
Supplement: Inserts. — Insert N1 to N3 Covid and Web. [file agh-88-1-3776-s1.zip › s1-agh-3776_reis/Insert_1.pdf]

Insert N1 Covid and Web. The early occurrence of cases in France and a later superspreading event reported by general media

Several pieces of information issued from French general media in May 2020 without any confirmation, e.g., publications in scientific journals pointing to an early occurrence of COVID-19 cases in fall 2019 (1,2) and the Alsatian superspreading event in March 2020 (3).

1.www.fondation-diaconat.fr/images/Presse/2020/CP-HAS-Imagerie-mdicale-7-mai-2020.pdf. . Access online March 11, 2022

2.www.letelegramme.fr/local/cotes-d-armor/covid-19-une-costarmoricaine-aurait-ete-contaminee-fin-novembre-en-france-15-05-2020-12552890.php. Access online March 11, 2022

3 www.leparisien.fr/societe/au-coeur-des-clusters-a-mulhouse-le-rassemblement-evangelique-qui-a-fait-basculer-la-france-09-05-2020-8313693.php. Access online March 11, 2022
