# Supplementary material for: Covid-19: Early Cases and Disease Spread
Source: Ann Glob Health. 2022 Sep 29;88(1):83. doi: 10.5334/aogh.3776 (PMC9524236; doi:10.5334/aogh.3776)
Supplement: Inserts. — Insert N1 to N3 Covid and Web. [file agh-88-1-3776-s1.zip › s1-agh-3776_reis/Insert_2.docx]

**Insert N2 Covid and Web, Chinese general media**

Three important articles issued in the general media. The first one is about an early COVID-19 case in Wuhan (1). The second is the report of the CCDC director's declaration, Prof G.F Gao who excluded the Huanan Wholesale Seafood Market as the place at the origin of the pandemic in a statement of the China Global Television Network (2). The third announces an analysis of 200,000 serum samples identified by the WHO, collected in 2019 and stored in the Wuhan Blood Center (3).

1*.South China Morning Post* March 13, 2020. www.scmp.com/news/china/society/article/ 3074991/ coronavirus-china-first-confirmed- covid-19-case-traced-back. Access online March 11, 2022

2.China Global Television Network. https://news.cgtn.com/news/2020-05-26/Official-Wuhan-seafood-market-may-be-the-victim-of-the-coronavirus--QNVxtMlFbW/index.html. Access online January 31, 2022

3. Walsh NP, China to test thousands of Wuhan blood samples in Covid-19 probe. www.cnn.com/2021/10/12/asia/china-wuhan-blood-samples-covid-19-probe-intl-cmd/index.html. Access online January 31, 2022
