# Supplementary material for: Covid-19: Early Cases and Disease Spread
Source: Ann Glob Health. 2022 Sep 29;88(1):83. doi: 10.5334/aogh.3776 (PMC9524236; doi:10.5334/aogh.3776)
Supplement: Inserts. — Insert N1 to N3 Covid and Web. [file agh-88-1-3776-s1.zip › s1-agh-3776_reis/Insert_3.docx]

**Insert N3 Covid and Web, COVID-19 and the CISM Military World Games in Wuhan, 18-28 October 2019, general media reports.**

Geopolitical issues have blurred facts leading to manipulations and withholding of information, in order to create an official story telling. This is probably the case of the "*five American athletes"* (38) hospitalized first in Wuhan Jin Yinan Hospital during the Games, with the diagnosis of malaria. The "Polygraph.info", a fact-checking website produced by Voice of America (VOA), has pointed to two errors (1)​. The diagnosis of malaria rises serious doubt as China was certified malaria-free for four years by the WHO in mid-2021. Second the citizenship of the athletes was never confirmed. and it seems that at least two athletes came from Africa (1). Sparse information is available regarding the athletes who returned home ill from Wuhan. In March and later in May 2020, as the Summer Olympic games approached, several athletes from France (2), USA (3), Italy (4), Spain (5), Luxembourg (6) and other European country participants of the World Military in Wuhan (78), declared that they complained of having been ill during the Military World Games in October 2019 or upon their return from Wuhan. They evoked *a posteriori* a diagnosis of COVID-19. Several general media covered this concern for some months, but unhappily no scientific investigations have been conducted.

The French Ministry for Armed Forces denied the declarations of the French Athletes who were never officially tested (6). In Spain, the Ministry of the Army tested 138 military athletes; the results, which became public in June 2020, showed that 6 participants were positive for SARS-CoV-2 antibodies, without details (7). Official statements from military authorities from France and Switzerland range from denial to reassuring phrases (8). Commenting in May 2020 on these statements, Sweden's chief epidemiologist Anders Tegnell declared that Covid-19 cases had probably occurred in November 2019 (9).

1.Echols W, Zhang L. Here We Go Again: U.S. Military Did Not Bring COVID-19 To Wuhan. www.polygraph.info/a/military-world-games-us-army-wuhan/31417109.html. Access online March 11, 2022

2.Anonymous. Une athlète ligérienne pense avoir été atteinte du Covid à Wuhan lors des jeux militaires en octobre, l'armée s'étonne. https://france3-regions.francetvinfo.fr/auvergne-rhone-alpes/loire/saint-etienne/coronavirus-athlete-stephanoise-affirme-avoir-ete-contaminee-octobre-wuhan-lors-jeux-mondiaux-militaires-1825160.html. Access online March 11, 2022

3.Squitieri T. Did the Military World Games Spread COVID-19? https://prospect.org/coronavirus/did-the-military-world-games-spread-covid-19/. Access online March 11, 2022

4.Bonarrigo M. Tagliariol: “*Malato dopo i Giochi militari a Wuhan. Coronavirus? Non so, ma l’infezione era ai polmoni*». www.corriere.it/sport/20_maggio_07/tagliariol-dopo-wuhan-mi-sono-ammalato-l-infezione-ha-colpito-polmoni-209e7f6a-9049-11ea-b981-878bbbd902eb.shtml. Access online March 11, 2022.

5. Aguilar-Amat D. El Covid-19 llegó a España en octubre con los Juegos Militares de Wuhan: "*Tuve todos los síntoma*s". www.elespanol.com/deportes/otros-deportes/20200517/covid-19-espana-octubre-juegos-militares-wuhan-sintomas/490701382_0.html. Access online March 11, 2022

6. CNEWS. COVID-19 : des athlètes français contaminés dès octobre aux jeux militaires ?

www.cnews.fr/monde/2020-05-18/covid-19-des-athletes-francais-contamines-des-octobre-aux-jeux-militaires-958202. Access online March 11, 2022

7. Redacción. Seis de 138 militares de los juegos de Wuhan dan positivo a anticuerpos Covid.

www.lavanguardia.com/politica/20200619/481840336786/seis-de-138-militares-de-los-juegos-de-wuhan-dan-positivo-a-anticuerpos-covid.html. Access online January 31, 2022

8. Anonymous. Coronavirus : Des athlètes romands présents à Wuhan témoignent. www.lematin.ch/story/des-athletes-infectes-a-wuhan-en-octobre-deja-990586772177. Access online March 11, 2022

9. Stickings T and Cachia A. Sweden probably had Covid-19 cases in November, country's virus chief claims, after French man is found to have been infected in December. www.dailymail.co.uk/news/article-8288599/Sweden-probably-Covid-19-cases-NOVEMBER-countrys-virus-chief-claims.htm. Access online March 11, 2022.
